# Investigating the impact of extraneous distractions on consultations in general practice: Lessons learned

**DOI:** 10.1186/1471-2288-9-8

**Published:** 2009-02-05

**Authors:** Moyez Jiwa, Robert McKinley, Carolyn O'Shea, Hayley Arnet, Katrina Spilsbury, Marthe Smith

**Affiliations:** 1 Western Australian Centre for Cancer and Palliative Care, Curtin Health Innovation Research Institute, Curtin University of Technology, Perth, Western Australia, Australia; 2 Department of General Practice, Keele University School of Medicine, Keele University, Keele, UK; 3 Department of General Practice, Monash University, Melbourne, Victoria, Australia; 4 Centre for Population Health Research, Curtin Health Innovation Research Institute, Curtin University of Technology, Perth, Western Australia, Australia

## Abstract

**Background:**

Extraneous distractions may influence the flow of general practice consultations. This study piloted a methodology to examine the impact of interrupting general practitioners (GPs) while consulting actor-patients.

**Methods:**

Six GPs were video recorded consulting six actor-patients each presenting a different clinical scenario in a simulated surgery. Five cases presented red flag cancer symptoms. Half the consultations were interrupted. Two independent assessors, blinded to the occurrence of interruptions, assessed consultation performance using the Leicester Assessment Package (LAP) for clinical competence.

**Results:**

24 of 36 consultations were video recorded with sufficient audio-visual clarity to allow scoring. The association between LAP score and three variables could be studied: a variety of interruptions, different GPs and various scenarios. Agreement between assessors on GP performance was poor and showed an increased bias with increasing LAP score. Despite this, the interruption did not significantly impact on assessor LAP scores (Mean difference: 0.22, P = 0.83) even after controlling for assessor, different GPs and scenarios.

**Conclusion:**

Extraneous distractions had no impact on GP performance in this underpowered pilot study, a conclusion which needs to be confirmed in a larger study. However several important lessons were learned. Recorded actor-patient clinical sessions are logistically challenging. GPs whose skills were not previously assessed were working in unfamiliar surroundings dealing with relatively straight forward diagnostic challenges and may have anticipated the interruptions. In a redesign of this experiment it may be possible to eliminate some of these limitations.

## Background

In Australia, as in many other countries the general practitioner (GP) is usually the first medical practitioner to consult patients with any significant health care problem. The core activity which takes place in general practice is the consultation. The function of the consultation has remained essentially unchanged over many decades and the description by the British Royal College of General Practitioners in 1972 still has resonance today:

*"...the ideal consultation. The doctor's attention is devoted exclusively for a short period of time to the life and problems of another human being. He is there to listen and to help. His training will have made him receptive to a wide range of distress signals and given him the means, to answer them. The occasion will be unhurried and something will be gained by both participants; a good consultation brings satisfaction to the doctor as well as to the patient *[1]."

Many factors may affect the interaction between doctor and patient they include interruptions, new technologies or alterations to established routines. Although there are no published Australian data, one United Kingdom general practice study found that 10% of consultations were interrupted, mostly by phone calls (49%) and staff (38%) [2]. Another study in the United Kingdom found 6.4% of consultations interrupted by the telephone and 1.9% by a person [3].

Interruptions affect both the patient and doctor. Twenty percent of patients in one study reported that interruptions had a 'bad effect' on their consultation and 40% report that the consultation 'would have been better' without the interruption [2]. Eighty four percent of Israeli primary care physicians reported that interruptions are harmful and disruptive to consultations and 92% that interruptions have a negative influence on the doctor-patient relationship [4]. Interruptions are a source of stress to doctors in the UK and Spain [5,6]. Emergency department medical and nursing staff identify interruptions as a distraction that contributes to deficiencies in management of clinically significant events [7]. Therefore a study in general practice confirming the adverse impact of interruptions on core competencies may lead to changes in the way practitioners organize their practice to enhance patient safety and satisfaction.

It would be unethical to perform an experiment by interrupting 'real' consultations when there are data indicating that interruptions cause harm. However if we are to frame a study of interruptions within a simulated clinical environment a number of variables must be taken into account. They include general practitioners, clinical problems, interruptions and the application of a reliable and valid outcome measure. In detail these variables include:

1. The different consultation style and competencies of the practitioners.

2. The impact on doctor/'patient' of being observed.

3. The impact of conducting the study in surroundings other than the doctors' normal consultation rooms.

4. The timing and nature of interruptions.

5. The nature of the problems being presented.

6. The authenticity of the presentation by the actor-patient consulting multiple doctors portraying the same scenario.

7. The application of reliable and valid measures and the stringency of scoring by the assessors.

## Methods

We aimed to explore the impact of interruptions on GP consultation performance. To do this we had to develop and pilot a methodology. Our objective was to compare performance on six consultation competencies with and without interruptions

### Actor-patients

Five of six 'patients' presented with a red flag symptom of common cancers [8]. The symptoms (Table 1) were readily recognizable as those of a cancer with a detailed history. Physical signs, presented as photographs or descriptions, were available if the GP proposed a relevant examination.

**Table 1:** Cancers

Diagnosis: Signs and symptoms
1. CNS cancer: Recurrent headaches of 4 months duration with neurological symptoms on examination

2. Prostatic Cancer: Prostatic symptoms (Description of irregular, hard prostate gland offered when patient examined)

3. Lung cancer: Haemoptysis

4. Breast Cancer: Breast lump

5. Non-cancer patient: Presenting for repeat prescription of asthma medication

6. Colorectal cancer: Anaemia, with change in bowel habit for 9 weeks duration

Actors were members of staff at the research centre in Western Australia. They were instructed to present as patients consulting for ongoing care (Table 2) and to mention a new problem during the consultation. A medical record was prepared for each patient and was available to the GP.

**Table 2:** 'Ticket of entry' to consult. The sixth case was a non-cancer case.

"Ongoing care" problem	Request or task
Hypertension	Request for repeat prescription of hypertension medication and review of stable hypertension.

Migraine	Patient sought a repeat prescription for migraine prophylaxis. This case presented with classic symptoms of a CNS tumour, volunteered after closer questioning of change in symptoms since last consult.

Diabetes	Review of diabetes. Patient was concerned about a potentially infected abrasion.

Breast check and PAP smear	Patient presented for PAP smear and breast check.

Blood results	Patient attending to get results of full blood count ordered at previous consultation for fatigue.

### Interruptions

A list of consultation interruptions was generated by the research team. The interruptions selected were considered by the team to be common and potentially most disruptive. The interruptions (Table 3) and the consultations were randomly paired so that three of each GP's consultations were interrupted.

**Table 3:** Planned interruptions

Distraction	Detail
Mobile phone	The patient took a mobile phone call during the consult.

Receptionist	A receptionist asked the doctor for information from the patient files about another patient during the consult.

Car	The patient left the room for two minutes to deal with their vehicle in the car park.

### Consultations

Six volunteer GPs were asked to consult with the six simulated patients as though they had previously visited another GP at the surgery for one or two ongoing medical problems (e.g. diabetes, hypertension.etc). They had 15 minutes per consultation. GPs were asked to keep consultation notes and outline any management plan in as much detail as they would in practice. After two minutes, three of the consultations were interrupted for two minutes.

### Blinding

#### Participants

GPs were told that the study was about 'evaluating interruptions in consultations'. They were unaware of the nature or relevance of the interruptions to the presenting cases.

#### Observers

Video recordings were edited to remove the interruptions and reviewed by two associate investigators (RMK and COS) who were unaware of which study arm each consultation was from. All videos had an edit, but in only some of these edits was an interruption. The edit also always removed any cues any interruption, such as the phone ringing or a participant apologizing for the interruption. This allowed the assessors to remain blinded to the presence or absence of an interruption when assessing the tape.

### Outcome measures

#### Consultation competence

The LAP has been shown to facilitate reliable assessments of consultation performance and its face validity has been confirmed for general practice consultations [9-11]. Three of the six LAP categories of consultation competence (interviewing and history taking, problem solving and patient management) were assessed in this study. We double rated all available consultations and followed the methods described in the LAP and previous work on assessing recorded consultations [12-14]. A difference of 5 or more in the LAP scores was considered 'clinically significant'. This was based on a standard deviation of about 10 for LAP assessments of 53 GPs and a before and after difference of 5 points (unpublished data from a series of studies on GPs' consultation skills) [15,16].

## Results

### Scoring by two assessors

Only twenty four of the thirty six consultations were recorded with sufficient sound and picture quality to allow analysis using the LAP. Twelve consults could not be scored because of failures of recording equipment. The assessors confirmed after coding the videos they were unable to work out from the video which consults were or were not interrupted.

### Differences in LAP scores between assessors

**Figure 1:**
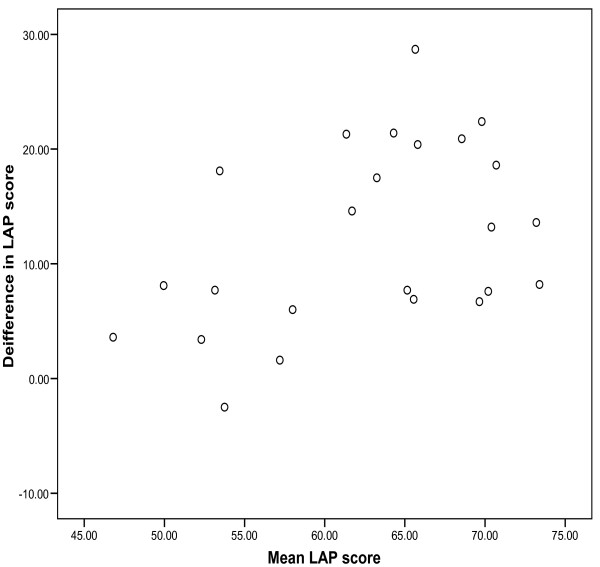
**Difference in LAP scores between assessors vs. mean LAP score**.

Agreement between the two assessors was explored by a Bland-Altman plot of the difference in LAP scores against the mean LAP score (Figure 1). The mean difference in LAP score was 12.3 (95%CI 8.9–15.7) with limits of agreement (mean ± 2SD) ranging from -3.7 to 28.3. Pitman's test of difference in variance, the correlation coefficient of the difference versus mean of the two measurements was 0.431 (p = 0.035). This indicates that the better the LAP score for the consultation the greater the difference between the two assessors. Overall 20 out of 24 LAP scores (83.3%) were scored with differences greater than 5 LAP points between the assessors. This confirms that one assessor was more conservative than the other.

### How did interruptions impact on GP performance?

There was no significant difference in the mean LAP scores for consultations with interruptions, 62.4 ± SD10.9 and without interruptions, 62.9 ± SD10.7 (t-test p = 0.891). In order to control for the impact of the different assessors, the clinical scenario and GP in the presence of correlated measures, a generalized estimating equations (GEE) approach was used. After controlling for other covariates, a mean difference in LAP scores of 0.22 (95%CI: -1.9–2.3) was observed between consultations with and without interruptions. In other words, the interruptions to the consultation did not impact on GP performance. However, this pilot study had only 80% power to detect a difference of 8 or more in mean LAP score with an alpha level of 0.05. This indicates that our conclusion of no difference between scores with or without interruption was subject to type II error. A much larger study would be needed to confirm whether the observed difference of 0.22 existed.

## Discussion

The approach had several strengths; we were able to control factors that may be difficult to control in clinical practice. The practitioners all consulted the same patients and were subject to similar interruptions occurring at similar points in each consultation. All practitioners were consulting in the same practice on the same evening. In many ways the methodology involving consulting actor-patients mimics the formal assessment or examinations of candidates seeking membership to many professional colleges. A further strength of the methodology was the ability to edit the consultations to allow the assessors to be successfully blinded as to the occurrence of the interruptions at analysis. However the application of this methodology did not demonstrate any effect of interruptions on the overall consultation performance of GPs, which may be a reflection of the small sample size and reduced power. This study however illustrates methodological and technical challenges of investigating the impact of interruptions on consultations in this context. A summary of the challenges and potential solutions is in Table 1.

### Medical practitioners

Participating GPs were volunteers and perhaps unrepresentative. That alone was not considered a major limitation in this study. However we have no measures of how the practitioners perform in their everyday practice using the LAP or any other consultation competence measure. We are therefore unable to confirm if their performance here reflects their competence in routine clinical practice. We therefore recommend a preview of practitioner performance in routine practice with 'live' patients in a future study to provide a practitioner performance baseline.

### The clinical challenge

Many more people present to general practitioners with symptoms which could indicate cancer, than people who actually have cancer: symptoms have a relatively low sensitivity and specificity. Conversely, forty percent of cancer patients reported significant problems communicating their concerns in the pre-diagnostic period and needed recurrent GP consultations before being 'taken seriously'[17]. We hypothesised that interruptions may explain some of this apparent inattentiveness of GPs and therefore planned interruptions to consultations, where cancer featured prominently on the list of differential diagnosis. In practice however the clinical encounters adopted for this study were fairly self evident. The presentations may consequently have led to ritualized practice in which practitioners reverted to well rehearsed questioning and examination routines. Indeed the GPs commented that the majority of the consultations involved cancer symptoms, which was unusual in reality and may have affected their performance. Neither practitioner examination skills nor the impact of examination performance on the consultation could be assessed in this experiment. However it may be impractical and possibly unethical to subject actors to physical examinations or tests. In this study it is also possible that there were subtle differences in the style of presentation which may have had an impact on practitioner performance. The employment of professional actors may have been an advantage. It may also be important that the study is conducted in a setting that more closely resemble the practitioner's rooms. It may be possible and even necessary to furnish the study 'clinic room' in consultation with the participating practitioners.

### The interruption

Firstly, informal feedback from participants suggested that some interruptions tested were considered 'routine' and irrelevant. Others, such as the patient taking a mobile telephone call, were thought more likely to upset the consultation flow. However we recommend that a list of possible interruption is formally researched with a wider group of practitioners. It would be prudent to systematically identify which are considered problematic as our study suggests that intuition may be misleading. The timing and duration of the interruptions was also standardized in this study. It is possible that interruptions are more problematic at different points in the consultation. Finally the information sheet for the study indicated that participants were invited to a study on 'evaluation of interruptions in consultations'. Although no details were provided the interruptions may have been anticipated.

### Blinding of assessors

The assessors according to retrospective self report were successfully blinded to the presence or lack of a consultation interruption. This was achieved by editing each video tape carefully to remove any cues to the possible presence or absence of an interruption. In future projects it would be useful to get the assessors to report as they assessed each tape whether they felt there had been any interruption, to provide a measure of how successful or not the blinding process had been.

### The scoring of consultation competencies

Recording of consultations needs to be facilitated by technicians guaranteeing high quality footage and with the least disruption or inconvenience to the participants. Unfortunately a relatively large proportion of consultation was not captured on tape and so could not be analysed. It may be helpful in a repeat of this study to employ a professional media team to facilitate the recordings. We were unable to assess the impact of observation on the GPs' performance although the literature on video recording for the purposes of assessment suggests that it has no significant adverse effect [18]. Agreement by assessors was generally quite poor. The assessors were from very different practice backgrounds (UK and Australia), experience as a GP (20 vs. 5 years), experience of assessment (15 vs. 3 years) and familiarity with the LAP (RMK) involved in the design, development and evaluation of the LAP, COS new to it). More resources need to be devoted to cross-training and calibration of the assessors. Nevertheless, we did not record any significant difference in the assessment of cases with reference to interruption impact. Also taken individually there was no trend in the individual scores for the assessors to show an appreciable effect of interruptions. However, this study was designed to investigate the practicalities of establishing the methodology rather than obtain conclusive results regarding the effect of interruptions. A much large study with more power is needed to confirm these results.

## Conclusion

Several important lessons were learned. Simulated consultation sessions are logistically challenging. GPs with unknown skills were working in unfamiliar surroundings dealing with relatively straight forward cases and may have anticipated the interruptions. Because of the need to control for a large number of variables in the study it may be difficult in research on interruptions to test their impact in practice within the setting of actual clinics with 'real' patients. Therefore there is a need for a 'clinical laboratory' that can be used to field test other interventions within controlled conditions. Such a laboratory could be used to test the impact of a variety of factors or innovations that can subsequently be refined and tested in other experimental designs. In this study we offer some preliminary ideas on the design of such a facility.

## Competing interests

The authors declare that they have no competing interests.

## Authors' contributions

MJ, RMcK, MS designed the study and co-authored the paper, CO'S, RMcK and KS analysed the data and co-authored the paper HA organized the simulated consults, collected the data and co-authored the paper. All authors read and approved the final manuscript.

## Pre-publication history

The pre-publication history for this paper can be accessed here:

http://www.biomedcentral.com/1471-2288/9/8/prepub
